# Thrombocytopenia in the ICU: disseminated intravascular coagulation and thrombotic microangiopathies—what intensivists need to know

**DOI:** 10.1186/s13054-018-2073-2

**Published:** 2018-06-13

**Authors:** Jean-Louis Vincent, Pedro Castro, Beverley J. Hunt, Achim Jörres, Manuel Praga, Jose Rojas-Suarez, Eizo Watanabe

**Affiliations:** 1 Department of Intensive Care, Erasme University Hospital, Université libre de Bruxelles, Brussels, Belgium; 2 Medical Intensive Care Unit, Hospital Clinic of Barcelona, IDIBAPS, University of Barcelona, Barcelona, Spain; 3 Thrombosis and Haemophilia Centre, Guy’s and St Thomas’ NHS Foundation Trust, London, UK; 4 Clinic for Nephrology, Transplantation Medicine and Intensive Care Medicine, University Witten/Herdecke Medical Centre, Cologne-Merheim, Germany; 5 Division of Nephrology, Instituto de Investigación Hospital 12 de Octubre (imas12), Complutense University of Madrid, Madrid, Spain; 6 Intensive Care Unit, Obstetric Medicine and Internal Medicine, Gestion Salud IPS Clinic, University of Cartagena, Cartagena, Colombia; 7 Department of Emergency and Critical Care Medicine, Eastern Chiba Medical Center, Togane City, Japan

**Keywords:** Disseminated intravascular coagulation, Hemolytic uremic syndrome, Intensive care unit, Thrombotic microangiopathy, Thrombotic thrombocytopenic purpura

Thrombocytopenia affects 25–55% of intensive care unit (ICU) patients [1]. The reasons for thrombocytopenia in the ICU are numerous, including, among others, sepsis, drugs, and the use of extracorporeal devices (Fig. 1) [1]. Some patients with thrombocytopenia also have microangiopathic hemolytic anemia (MAHA), accompanied by elevated serum lactate dehydrogenase levels and schistocytes on the blood film [2, 3]. This combination of thrombocytopenia and MAHA, in which thrombi form in the microvasculature and schistocytes develop from red cell destruction as they pass over these thrombi [2], occurs in patients with disseminated intravascular coagulation (DIC), but also in those with thrombotic microangiopathies (TMAs), including thrombotic thrombocytopenic purpura (TTP) and hemolytic uremic syndrome (HUS).

**Fig. 1:**
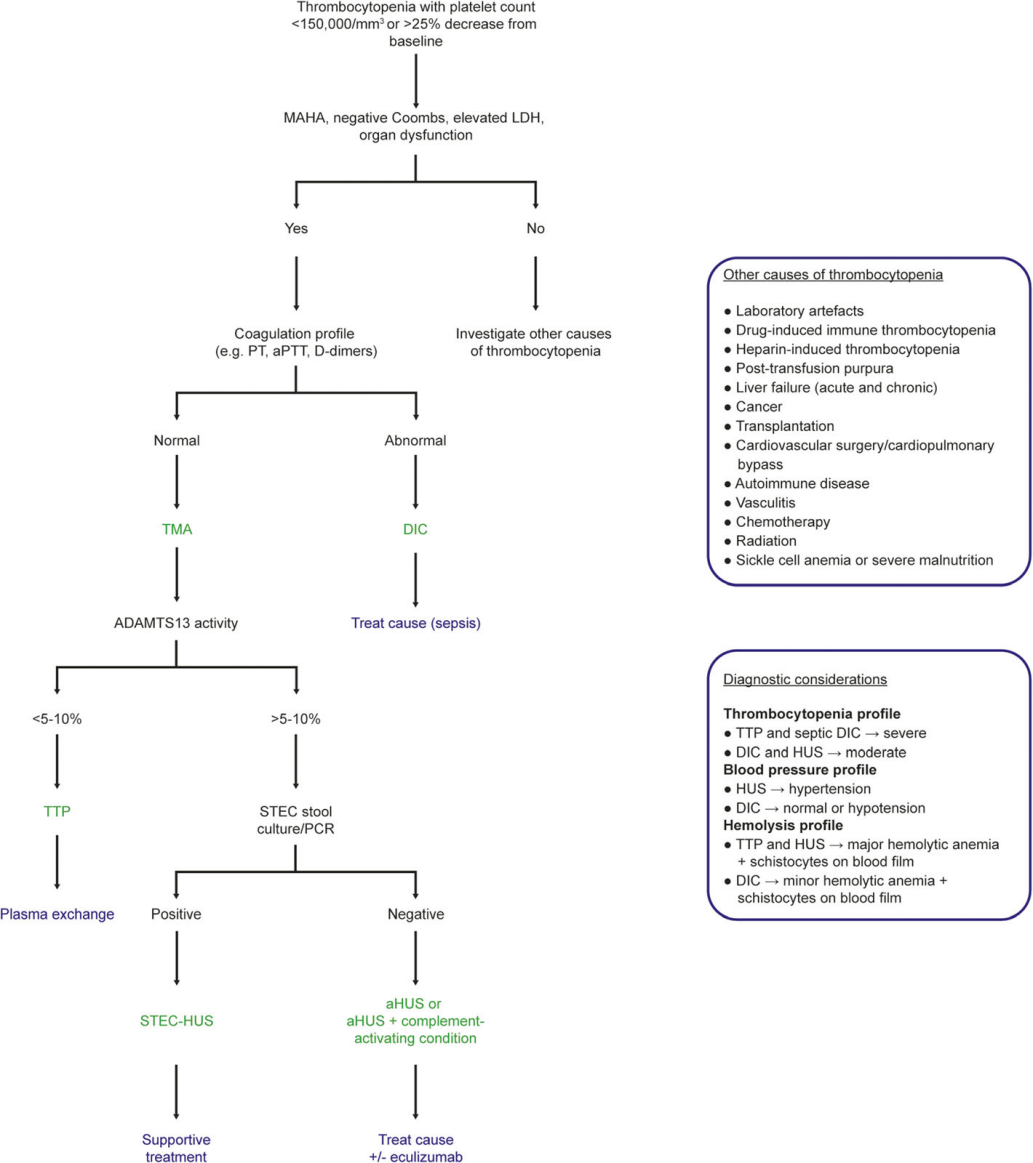
An algorithm to rapidly differentiate disseminated intravascular coagulation (DIC) from thrombotic thrombocytopenic purpura (TTP) and hemolytic uremic syndrome (HUS) in the intensive care unit (ICU). Thrombocytopenia with microangiopathic hemolytic anemia (MAHA), negative Coombs test, elevated lactate dehydrogenase (LDH), and organ dysfunction are common to DIC, TTP, and HUS. Abnormal coagulation studies, including prothrombin time (PT), activated partial thromboplastin time (aPTT), fibrinogen concentration, fibrin degradation products, D-dimers, and antithrombin, are required for differentiation of DIC from thrombotic microangiopathies (TMAs). Additionally, blood pressure should be considered because HUS usually presents with hypertension. Once DIC has been excluded, the underlying TMA must be identified. TTP is diagnosed by identification of low ADAMTS13 activity (< 5-10%) and treated urgently with plasma exchange initially; HUS is associated with normal ADAMTS13 activity (> 5–10%) and the type of HUS elucidated by performing a Shiga-toxin producing *Escherichia coli* (STEC) stool culture or polymerase chain reaction (PCR) assay. Positive STEC strongly suggests STEC-HUS; negative STEC strongly suggests aHUS, with or without an associated complement-activating condition (e.g., infection, malignant hypertension, the post-partum period, kidney transplantation, drugs, or malignancy). Rapid detection and management of any associated complement-activating condition and consideration of eculizumab are required [3, 6, 9, 13]

DIC is relatively common, developing in 9–19% of ICU patients, usually as a result of sepsis [4], with an incidence of 18/100,000 in the overall population [2, 5]. By contrast, TTP and Shiga-toxin producing *Escherichia coli* (STEC)-associated HUS have estimated incidences of 6 and up to 29 cases per million, respectively, and atypical HUS (aHUS) a prevalence of 0.2–0.4 cases per million [6, 7], making these conditions far rarer than DIC. Although TTP is described as a pentad of fever, thrombocytopenia, MAHA, renal dysfunction, and neurological impairment, often some of these features are not present [7]. Accordingly, TTP may be confused with HUS, which is most commonly characterized by the triad of thrombocytopenia, MAHA, and renal dysfunction [3]. These clinical similarities of DIC, TTP, and HUS are a major concern because they pose a risk of misdiagnosis as intensivists are more likely to consider a diagnosis of DIC than of the rarer TTP or HUS, thus delaying potentially lifesaving treatment.

Several diagnostic algorithms for TMA have been published [3, 8–10]. However, currently the only available guidance specific to the ICU are the recently published expert statements of Azoulay and colleagues [11]. This publication provides an excellent guide for the differential diagnosis of TMAs but only briefly mentions DIC. A concise diagnostic algorithm tailored to intensivists would aid rapid differential diagnosis of TTP and HUS from DIC, and enable early appropriate treatment.

## A new algorithm to rapidly differentiate DIC from TTP and HUS in the ICU

Given the importance of differentiating DIC from TTP and HUS, we propose a concise algorithm based on existing guidance [3, 9, 11] and our own discussions which will enable the intensivist to rapidly distinguish between these entities (Fig. 1). MAHA, negative Coombs test, elevated lactate dehydrogenase (LDH) levels, and organ dysfunction with thrombocytopenia are common to DIC, TTP, and aHUS [2, 3], although patients with TTP and septic DIC may have more severe thrombocytopenia [2, 12]. The most important distinguishing factor between DIC and TMAs is the coagulation profile, as patients with DIC have altered coagulation [2]. However, blood pressure is also important as HUS often presents with severe hypertension and DIC with hypotension [3, 7]. The combined evaluation of full blood count and blood smear, hemolysis profile, coagulation profile, and blood pressure is usually sufficient to ascertain whether a patient has DIC or a TMA.

Once DIC has been excluded, confirming the cause of the TMA is paramount for appropriate management. The two most concerning causes of TMA are TTP and HUS. TTP is caused by a deficiency in a disintegrin-like metalloproteinase with thrombospondin motif type 1 member 13 (ADAMTS13) and has 90% mortality without plasma exchange [7]. HUS is caused by either Shiga toxin (STEC-HUS) or complement dysregulation as a result of genetic predisposition or autoantibodies (aHUS) [3, 6, 7, 11]. An ADAMTS13 activity of < 5–10% is sufficient to confirm TTP [3, 9] and a positive Shiga-toxin stool culture or polymerase chain reaction (PCR) assay confirms STEC-HUS [3, 9]. In the absence of low ADAMTS13 levels and Shiga-toxin, aHUS, a rare but devastating TMA, is highly likely [6]. Similar to DIC, aHUS has a rapid onset and non-specific presentation [2, 3]. aHUS can be found in association with other complement-activating states such as infection, malignant hypertension, the post-partum period, kidney transplantation, certain drugs, or malignancies [3]. There can be substantial overlap in the presentation of these conditions and they may coexist with complement-mediated aHUS, making distinction difficult [3]. It should also be remembered that aHUS can present with malignant hypertension, which itself can cause TMA [6, 9]. Rapid diagnosis and treatment are essential to prevent irreversible organ damage and death [13].

Like any pragmatic guidelines, we chose to focus on the most common presentation as we considered this of most benefit. For comprehensive guidance on TMA diagnosis and management, we refer to other works, such as those of Scully et al. [7], Campistol et al. [3], Laurence et al. [9], and Azoulay et al. [11]. While the proposed algorithm applies to the majority of cases of thrombocytopenia, it must be noted that clinical judgment and collaboration with experts is essential, as exceptional clinical presentations do occur [14, 15].

It should also be noted that some of the tests required in the differential diagnosis (e.g., ADAMTS13 activity assay) are not available at all institutions. If rapid ADAMTS13 testing is not possible, the PLASMIC score, a seven-component prediction tool that can accurately and reliably predict the probability of severe ADAMTS13 deficiency [10], can be used. Additionally, we have not included genetic testing for the complement abnormalities of aHUS in our algorithm; while these can confirm an already suspected diagnosis of aHUS, the turnaround time is currently considerable and should not be relied upon in the ICU [11].

Critically ill patients have a range of clinical problems, including multi-organ failure, sepsis, and shock [5], and early diagnosis and management are crucial to optimize outcomes. We present a concise diagnostic algorithm that enables intensivists to make a rapid diagnosis so that they can initiate early appropriate management for ICU patients with thrombocytopenia. This algorithm adds to the current literature available to the intensivist [11], with a focus on differentiating TTP and HUS from DIC.

## Abbreviations

ADAMTS13: A disintegrin-like metalloproteinase with thrombospondin motif type 1 member 13
aHUS: Atypical hemolytic uremic syndrome
DIC: Disseminated intravascular coagulation
HUS: Hemolytic uremic syndrome
ICU: Intensive care unit
LDH: Lactate dehydrogenase
MAHA: Microangiopathic hemolytic anemia
PCR: Polymerase chain reaction
STEC: Shiga-toxin producing *Escherichia coli*
TMA: Thrombotic microangiopathy
TTP: Thrombotic thrombocytopenic purpura
